# Epimedium protects against dyszoospermia in mice with *Pex3* knockout by exerting antioxidant effects and regulating the expression level of P16

**DOI:** 10.1038/s41419-021-04435-8

**Published:** 2022-01-20

**Authors:** Haiyang Zhao, Tingting Zhao, Jihong Yang, Qianqian Huang, Hua Wu, Yueyun Pan, Hui Wang, Yun Qian

**Affiliations:** 1 Department of Histology and Embryology, Nanjing Medical University, Nanjing, China; 2 Experimental Teaching Center of Basic Medicine, Nanjing Medical University, Nanjing, China; 3 Reproductive Medicine Center of the Second Affiliated Hospital of Nanjing Medical University, Nanjing, China; 4 First School Of Clinical Medicine, Nanjing Medical University, Nanjing, China; 5 State Key Laboratory of Reproductive Medicine, Nanjing, China

**Keywords:** Senescence, Embryology, Infertility, Experimental models of disease, Translational research

## Abstract

Oxidative stress (OS) is one of the primary factors leading to male infertility. Oral administration of antioxidants has thus far been found to significantly improve the quality of human sperm. Therefore, antioxidant treatment has become the consensus among international experts on male infertility. In this study, peroxisomal biogenesis factor 3 (*Pex3*)-knockout (KO, −/−) mice were used as a model to compare the efficacy of three types of traditional Chinese medicine (TCM) granules (Epimedium [YYH], Cuscuta [TSZ], and Rhodiola [HJT]) for male reproductive function rescue. YYH was revealed to be the best and exerted a rescue effect on *Pex3*−/− mice with spermatogenesis defects. In addition, YYH prominently reduced ROS levels in the testes, inhibited DNA oxidative damage in spermatogenic cells, promoted the proliferation of spermatogenic cells, and inhibited apoptosis in *Pex3*−/− male mice. Furthermore, the mechanism by which YYH ameliorated dyszoospermia was confirmed via the establishment of cyclin-dependent kinase inhibitor 2 A (P16Ink4a)-KO mice. Specifically, *Pex3*−/− mice produced elevated amounts of ROS, which damaged germ cell DNA and further activated the signaling pathway of the cell senescence regulatory protein P16-CDK6, resulting in cell cycle arrest and eventually contributing to spermatogenesis dysfunction. YYH supplementation partially corrected the associated phenotype in gene KO mice by affecting P16 expression levels, thus improving the reproductive outcome to a certain extent.

## Introduction

Infertility is a global issue. WHO estimates that 10–15% of childbearing couples are infertile, and 50% of infertility cases are caused by male factors. Among infertile men, 30–80% have excessive production of reactive oxygen species (ROS) and decreased antioxidant capacity for various reasons, which ultimately lead to male infertility [1, 2].

Generally, oxidative stress (OS) is defined as an imbalance between the cell antioxidant defense system and the production of ROS [3]. OS is theorized to be one of the prominent biological molecular mechanisms of aging. Oxidative damage to the reproductive system is quite similar to male reproductive aging, and germ cells are particularly sensitive to OS with increasing age [4–7]. Thus, the disorder of the antioxidant system leads to a significant decline in the quality of germ cells with aging.

Previous studies have revealed that oral antioxidant treatment can remarkably improve sperm quality, elevate the live birth rate from 5% to 10–30%, and enhance the clinical pregnancy rate from 6% to 11–28%. In addition, antioxidant treatment has been established as the consensus treatment among international experts on male infertility [8, 9]. In early studies, animal models such as aging, obesity, d-gal, triptolide, cyclophosphamide, and radiation models were widely used to assess antioxidant treatment methods [10–13]. For example, an aging mouse model indicated that melatonin supplementation could reduce the occurrence of abnormal spindle morphology and oocyte aneuploidy [14]. Moreover, in recent years, many mouse models with genetic defects associated with reproductive dysfunction phenotypes have been used in the field of reproductive medicine [15–17]. The use of genetically deficient mice as model animals has become a clinical trend [18, 19]. For instance, a *Bmi1*-KO mouse model has been used to prove that Bmi1 deletion can activate P16/P19 signaling and promote OS damage, ultimately leading to reproductive dysfunction. Supplementation with the antioxidant *N*-acetyl-l-cysteine (NAC) can ameliorate the above outcomes [20]. All of these findings suggest that mice with antioxidation-related gene defects and reduced fertility can be used to evaluate the efficacy of antioxidants and to screen and study the effects of certain age-related antioxidants.

Traditional Chinese medicines (TCMs) can remarkably ameliorate male infertility [21]. Among them, Epimedium, Cuscuta, and Rhodiola have been used in the treatment of infertility for more than 2000 years and are extensively distributed in Asia, Europe, and other areas of the world [22–24]. Most related studies have focused on their pharmacological effects, such as their ameliorative effects on cardiovascular disease, osteoporosis, nervous system function, and aging [25–27]. In male reproduction, these TCMs can regulate reproductive endocrine function, promote sexual function, ameliorate spermatogenesis dysfunction, elevate sperm quality, adjust the hypothalamic-pituitary-testicular axis, improve erectile function, and exert other effects. The underlying mechanisms generally involve ROS and aging-related DNA damage [28–30]. However, it is not clear whether the specific mechanisms involve amelioration of OS-induced spermatogenesis in males via exertion of antioxidant effects and depletion of DNA damage.

Peroxisomal biogenesis factor 3 (PEX3) is a key protein in peroxisomal membrane synthesis that is present in all germ cells of the germinal epithelium [31] and can affect male reproductive function by regulating OS levels. One of the main functions of peroxisomes is to degrade ROS and thereby prevent cells from producing too much ROS through OS [32]. Peroxidase widely exists in spermatogenic cells of the testes as well as in testicular Sertoli-Leydig cells [33]. We found that *Pex3*−/− mice had abnormal sperm, male infertility, and increased testicular ROS levels but that the overall functioning of their other organs was completely normal. This finding indicated that *Pex3*−/− mice may be a good model for research on male reproductive function and the mechanisms of antioxidants. Therefore, in this study, *Pex3*−/− mice were used as a model to study whether TCMs such as Epimedium [YYH], Cuscuta [TSZ], and Rhodiola [HJT] could ameliorate spermatogenesis dysfunction in mice. In further mechanistic studies, we deleted *P16* (INK4a), a key gene in the age-related P16-Rb signaling pathway [34–36], in mice and constructed multigene deletion mice to further explore the specific mechanism of YYH in the amelioration of male aging-related reproductive dysfunction.

## Materials and methods

### Experimental design

Three types of TCM granules (Tianjiang Pharmaceutical Co., Ltd., China) were selected in this study. The doses of administration were based on the conversion of the clinical doses of the drugs from humans to mice [37].

### Animals

All mice were raised in a specific pathogen-free environment. All animal protocols were approved by the Animal Care and Use Committee of Nanjing Medical University. *Pex3* and *P16*-KO mice were gifts from the research teams of Sha Jiahao and Miao Dengshun from the State Key Laboratory of Reproductive Medicine, Nanjing Medical University. All the animals were divided randomly into groups with no <10 mice in each group, as shown in Figure Legends. The mice were fed to the 9th week after birth, and then were administered continuously for 45 days by intragastric administration. During this period, we observed the mice every day, and directly eliminated the mice with the abnormal physiological states. The breeding methods for the mice with complex gene deletion are detailed in Figure S1. DNA was extracted from the tails of progeny mice, and the genes were amplified with specific primers. The genotypes of the mice were identified by DNA sequence analysis.

The primer sequences used were as follows:

*Pex3* primer 1: 5′-GCCAAACCATAGCACCAGC-3′

*Pex3* primer 2: 5′-CTTTGTCCTCTTTCTGGGCAC-3′

*Pex3* primer 3: 5′-TCGTGGTATCGTTATGCGCC-3′

*P16* primer 1: 5′- GGCAAATAGCGCCACCTAT-3′

*P16* primer 2: 5′-GCCGCTGGACCTAATAACTTC-3′

*P16* primer 3: 5′-GACTCCATGCTGCTCCAGAT-3′

### Body weight and reproductive organ weight

The body weights of all of the experimental mice were measured weekly until the end of the experiment. The organ coefficients were calculated with the equation [(organ weight/body weight) × 100%].

### Biochemical analysis of antioxidant status

Frozen testis tissue was homogenized in ice-cold saline (testicular tissue weight:saline weight = 1:9) for 20 minutes to prepare a 10% (w/v) homogenate. Then, the homogenates were filtered and centrifuged at 3500 rpm and 4 °C for 15 minutes using a refrigerated centrifuge, and superoxide dismutase (SOD) (S010M, Beyotime, China) and glutathione peroxidase (GSH-Px) (S0053, Beyotime) activity and malondialdehyde (MDA) (S0131, Beyotime) levels in the supernatant were determined using a microplate reader. The assay results were normalized to the protein concentration in each sample (P0010, Beyotime) and are expressed as U mg^−1^ protein or nmol mg^−1^ protein.

### ROS and apoptosis analysis

Frozen sections of testicles were stained with a dihydroethidium (DHE) kit (R001, Vigorous Biol, China) according to the manufacturer’s instructions. Paraffin sections of testes were stained with a TUNEL staining kit (A113, Vazyme, China) according to the manufacturer’s instructions and then photographed with an LSM700 confocal microscope (Zeiss, Germany).

### Assessment of sperm motility and sperm count

For animal sperm preparation, the caudal epididymis from adult mice was dissected and cut. Then, the tissues were incubated in human tubal fluid (HTF) medium (EasyCheck, China) at 37 °C. The sperm samples were diluted, and a 10 μL aliquot of each sperm sample was evenly distributed on a glass chamber slide and analyzed using Computer Assisted Sperm Analyzer via the IVOS II™ system (Hamilton Thorne, Beverly, MA, USA).

### Analysis of the sperm malformation rate

To analyze the sperm malformation rate, 10 μL of fresh spermatozoa were placed on slides. The air-dried slides were fixed in 4% paraformaldehyde and stained with hematoxylin–eosin (HE). Fifteen microscopic fields were randomly selected. Then, all sperm were counted, and the total number of sperm in each mouse was calculated. A count of at least 200 sperm was required. Finally, the abnormal sperm were carefully examined, and the proportion of sperm with abnormalities in the head and tail was calculated (by two technicians).

### Histological analysis

Testes and epididymides were fixed overnight in Bouin solution (SLBJ3855V, Sigma). Then, the testes were dehydrated with an ethanol gradient. The testes were embedded in paraffin and cut into 5-μm-thick sections. The testicular sections were then dewaxed, rehydrated, and stained with HE (C0105s, Beyotime) and periodic acid-Schiff (PAS, 87007, Thermo). The seminiferous epithelium cycle stage of the testicular sections was determined according to morphological criteria. The staging was based primarily on the form and shape of the acrosome and to a lesser extent on the shape of the head of the sperm cell and the degree of chromatin condensation [38, 39].

### Immunohistochemistry and immunofluorescence analysis

For immunofluorescence staining, prepared mouse tissue sections were treated at 4 °C with anti-PEX3 antibodies (121215, Abcam; 1:200); later, the sections were incubated with a 488-conjugated secondary antibody (SA00013-2, Proteintech; 1:500). An LSM700 confocal microscope (Zeiss) was used for imaging.

For immunohistochemical staining, tissue sections were treated at 4 °C with anti-γ-H2A.x antibodies (9718 T, CST; 1:200). Then, the samples were incubated with the secondary antibody and colorized with DAB (DA1015, Solarbio, China). Immunohistochemical analysis was performed under a brightfield microscope (Zeiss Axioskop 2 Plus).

### Western blotting

Proteins were extracted with 8 M urea lysis buffer (8 M urea, 75 μM NaCl, 50 μM Tris-Cl pH 8.2) containing 1 mM phenylmethylsulfonyl fluoride and quantified using a Bradford Protein Assay Kit (Beyotime). The protein samples were separated in gradient gel and transferred to PVDF membranes (Millipore). The antibodies were as follows: anti-PEX3 (A7352, Abclonal; 1:1000), anti-P16 (211542, Abcam; 1:1500), anti-CDK2 (2546 S, CST; 1:1000), anti-CDK6 (3136 S, CST; 1:2000), anti-P19Arf (10272, Proteintech; 1:2000), anti-P53 (2524 T, CST; 1:1000), anti-GAPDH (60004, Proteintech; 1:2000), and anti-β-Actin (66009, Proteintech; 1:2000). The samples were incubated with the appropriate primary antibodies overnight at 4 °C after blocking in 5% nonfat milk. After washing three times in TBST, the samples were incubated with secondary antibodies at the appropriate dilutions for 2 h at room temperature. The results were recorded after enhanced chemiluminescence color development (Tanon, China). In this study, we used Image J software to calculate the gray value, and the ratio of the gray value of the internal reference protein in the same lane is regarded as the final result.

### In vitro fertilization (IVF) assay

Female C57B6 mice (6–8 weeks old) were injected with pregnant mare serum gonadotropin (5 IU, Ningbo Sansheng Pharmaceutical Co., Ltd., China) 48 h before human chorionic gonadotropin (HCG) (5 IU, Ningbo Sansheng Pharmaceutical Co., Ltd., China) treatment. After 16 h of stimulation with HCG, oocytes were collected in pre-equilibrated oocyte IVF medium. The oocytes in HTF medium (Easy Check) were mixed with the activated epididymal sperm, incubated at 37 °C at 5% CO_2_, placed in KSOM medium (Easy Check), and cultured undisturbed for 4 days. The developing embryos were identified microscopically based on progression to the blastocyst stage (*n* = 200).

### Assessment of fertility

To determine fertility, a male mouse was arranged to mate with two female mice. After normal vaginal plug formation was detected, the female mice were separated from the male mouse. The number of offspring produced by each female was recorded.

### Statistical analysis

All data analysis was carried out without distinguishing between experimental groups. All assays were repeated at least three times. SPSS 19.0, Excel, and GraphPad Prism 8.0. were used for statistical analysis. The statistical significance of differences in mean values was assessed with *t* tests (*P < 0.05; **P < 0.01; ***P < 0.001). Data points correspond to the mean of the independent experiments and error bars denote standard deviation.

## Results

### Verification and preliminary evaluation of the reproductive function of *Pex3*-KO mice

**Fig. 1:**
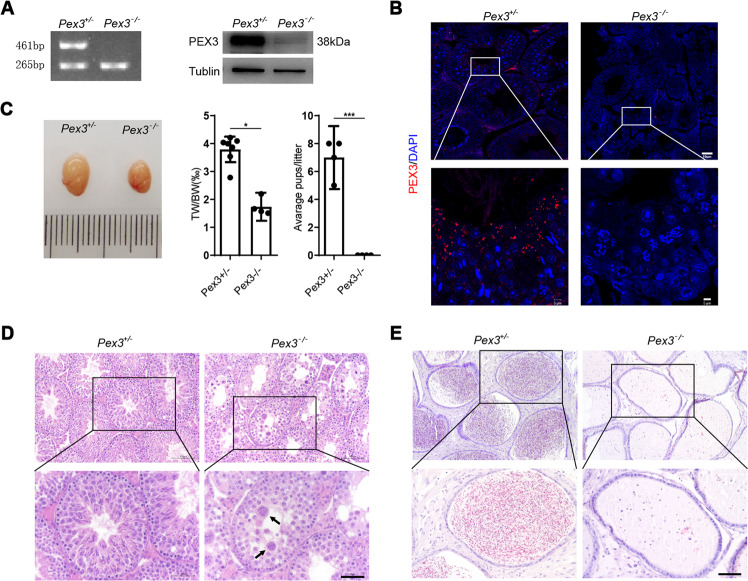
Phenotypic validation of *Pex3*-KO mice. (*n* = 4–7). **A** Mouse tail DNA agarose gel electrophoresis and testicular protein immunoblotting; tubulin was used as an internal control. **B** PEX3 immunofluorescence (red) staining of testicular tissue. Scale bars: 5 μm. **C** Representative testicular morphology, testicular weight ratio (TW/BW), and natural reproduction results. **D**–**E** HE staining of the testis and epididymis. Scale bars: 50 μm.

We eventually confirmed the successful construction of *Pex3*-KO mice (Fig. 1A/B), and the results of several phenotypic experiments showed that compared with wild-type male mice, *Pex3*-KO male mice had abnormal testicular development (Fig. 1C) and reduced testicular volumes and organ ratios and were completely infertile. Moreover, the results of HE staining of *Pex3*-KO mice revealed an abnormal testicular lumen structure (Fig. 1D). Sperm were completely absent from the epididymis (Fig. 1E), indicating spermatogenesis blockade. Unique pathological structures called syncytia [40] were present in the testicular lumen in *Pex3*-KO mice (arrow), which further proved the arrest of spermatogenesis.

### Different TCM supplements had different ameliorative effects on dyszoospermia in *Pex3*-KO mice, among which YYH had the best effect

**Fig. 2:**
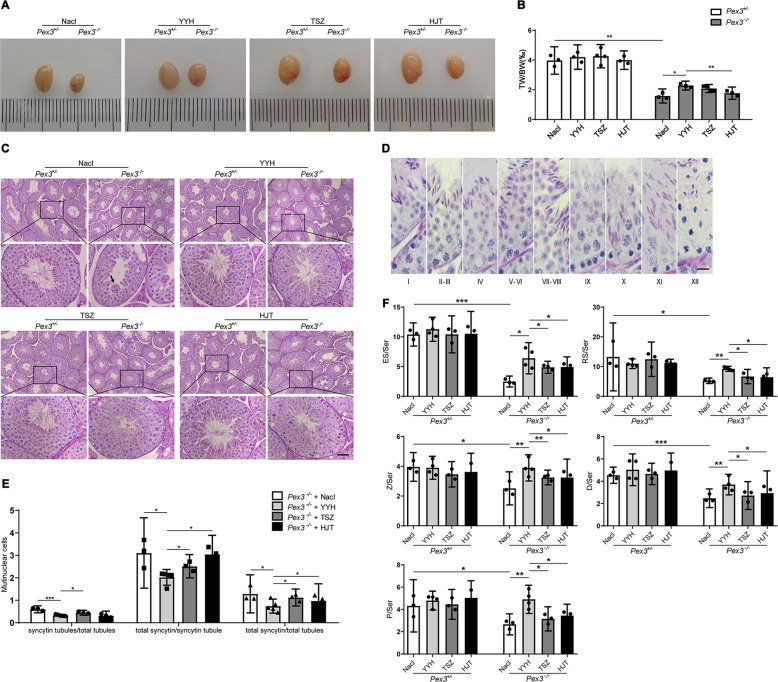
Morphological comparison of testes treated with three different TCMs. (*n* = 3–4). **A** Representative image of testicular sizes. **B** Testicular weight ratios. **C** HE staining of testes. Scale bars: 50 μm. **D** Criteria for distinguishing the time and phase of cells in the testicular lumen. Scale bars: 20 μm. **E** Statistics regarding syncytial multinucleated cells in the testicular lumen. **F** Sperm count statistics of each period. *ES* elongated spermatid, *RS* round spermatid, *Z* zygotene spermatocyte, *D* diplotene spermatocyte, *P* pachytene spermatocyte.

To investigate how to improve spermatogenesis in *Pex3*-KO mice, we selected three TCMs (TSZ, HJT, and YYH) for rescue experiments. A preliminary comparison revealed that YYH had the best effect. Subsequently, comparisons of testicular morphologies, sperm structures, and testicular seminiferous epithelial cycles revealed several findings. First, treatment of *Pex3*-KO mice with TCMs increased the testicular weight ratio and attenuated the abnormal testicular development (Fig. 2A, B). It also ameliorated dyszoospermia by increasing the number of elongated spermatids (Fig. 2D, F) and reduced the number of syncytial multinucleated cells so that testicular degeneration was ameliorated (Fig. 2C, E). YYH was more effective than TSZ and HJT. For the wild-type male mice, there was no significant change in any index with TCM supplementation. The above results implied that the three TCMs all improved the spermatogenesis disorder caused by *Pex3*-KO to a certain extent and the rescue effect of YYH was significantly better than those of the other two TCMs.

### YYH supplementation enabled *Pex3*-KO mice to produce transplantable embryos

**Fig. 3:**
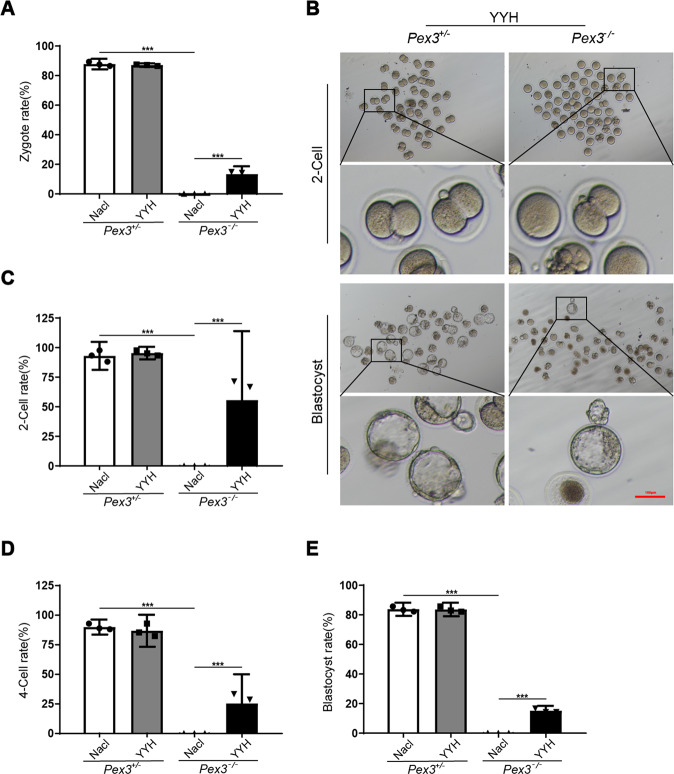
Effects of Epimedium granule supplementation on fertility outcomes and early embryonic development of *Pex3*-KO mice (*n* = 3). **A** Fertilization rate. **B** Representative contrast images of the *Pex3*+/− and *Pex3*−/− groups in the IVF experiment and blastocyst embryos in male mice after YYH administration. Scale bars: 100 μm. **C** Two-cell embryo rate. **D** Four-cell embryo rate. **E** Blastocyst rate.

After confirming that supplementation with YYH among the three TCMs significantly ameliorated spermatogenesis disorder in *Pex3*-KO mice, we further explored whether YYH supplementation had a rescue effect on the reproductive outcome. Consistently, the results indicated that YYH administration did not remarkably facilitate the ability of *Pex3*-KO mice to undergo natural pregnancy, but it increased the potential for obtaining early embryos. Early embryos were successfully obtained through IVF in the YYH-treated *Pex3*-KO (YYH-*Pex3*−/−) group (Fig. 3B), and the developmental ability of embryos was notably improved (Fig. 3A, C–E). Male NaCl-treated *Pex3*-KO (NaCl-*Pex3*−/−) mice could not undergo IVF due to the absence of sperm production. The above results demonstrated that although YYH supplementation did not improve the natural reproductive outcomes of *Pex3*-KO male mice, it enabled early embryos to be obtained, which made reproduction possible.

### YYH supplementation effectively improved OS in the testes of *Pex3*-KO mice

**Fig. 4:**
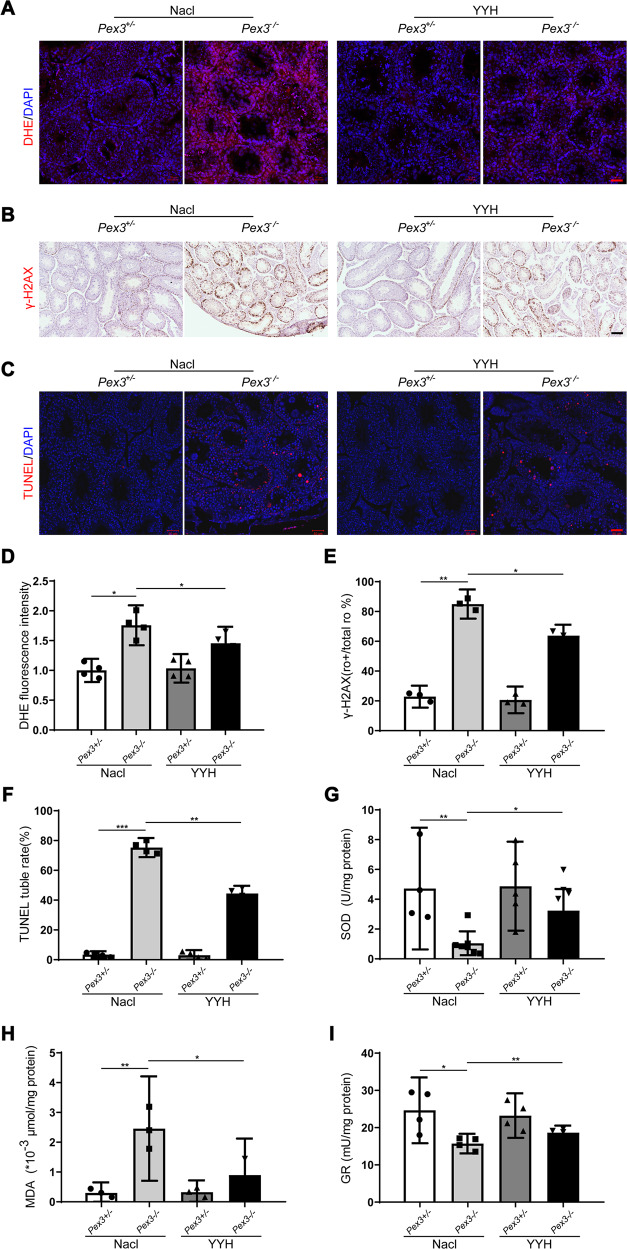
Effects of YYH supplementation on OS levels in testicular tissues of *Pex3*-KO mice. (*n* = 3–8). **A** DHE fluorescence (red) in testicular tissue from each group. Scale bars: 50 μm. **B** Images of γ-H2A.X immunohistochemical staining in testicular tissue from each group. Scale bars: 100 μm. **C** Image of TUNEL staining (red fluorescence) showing testicular apoptosis in each group. Scale bars: 50 μm. **D** DHE fluorescence intensity. **E** Rate of γ-H2AX-positive luminal cells. **F** Rate of TUNEL-positive luminal cells. **G** SOD level. **H** MDA level. **I** GR level.

Given the strong antioxidant function of YYH, we investigated whether YYH exerted a rescue effect by improving antioxidation in *Pex3*-KO mouse testes. We compared the levels of ROS (with the fluorescent probe DHE), DNA damage (via γH2A.X detection), apoptosis (via TUNEL staining), lipid peroxidation (via MDA detection), SOD activity, and glutathione reductase in the testicular tissues of male mice. The results revealed that compared with the NaCl-*Pex3*−/− group, the YYH-*Pex3*−/− group showed lower ROS levels (Fig. 4A, D); less DNA damage (Fig. 4B, E), apoptosis (Fig. 4C, F), and lipid peroxidation (Fig. 4H); and greater antioxidant capacity (Fig. 4G, I). However, the wild-type male mice displayed no apparent changes. The above results proved that YYH depleted OS injury by enhancing the antioxidant levels and reducing the ROS levels in spermatogenic cells. These changes reduced DNA oxidative damage and apoptosis in spermatogenic cells and ultimately enabled amelioration of spermatogenesis disorder and improvement of reproductive outcomes.

### YYH supplementation attenuated reproductive dysfunction in *Pex3*-KO mice by regulating the expression levels of proteins related to the cell senescence pathway

**Fig. 5:**
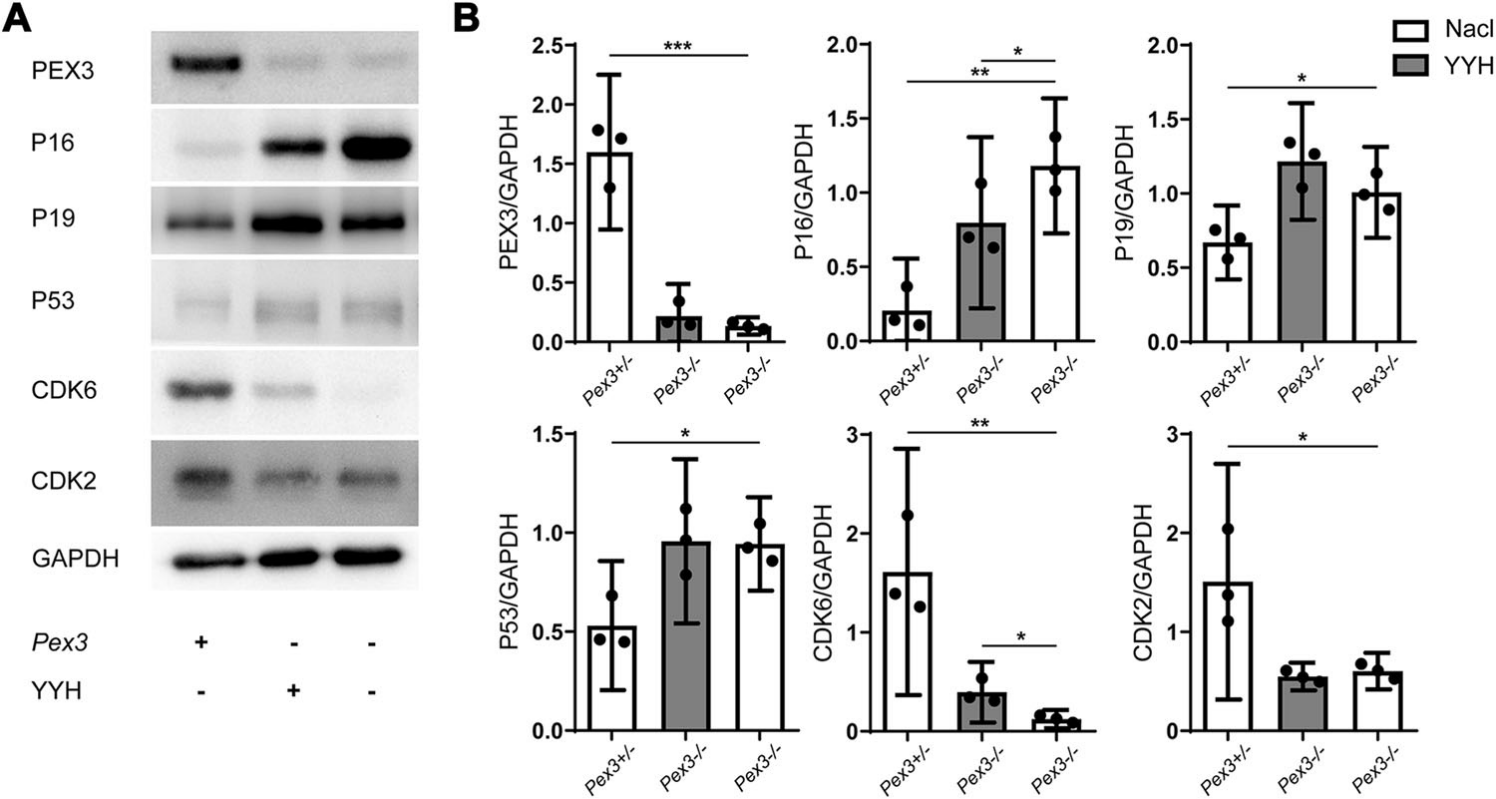
Detection of the expression levels of senescence pathway-related proteins after YYH supplementation. (*n* = 3). **A** Western blot results for P16-Rb and P53-P19 signaling-related proteins. **B** Grayscale values.

In this study, we found a series of testicular senescence-related phenotypes, such as increased testicular luminal cell apoptosis, DNA damage and ROS levels as well as spermatogenic cell degeneration. These findings implied that the senescence pathway was a good entry point to conduct in-depth studies on the mechanism of YYH [41, 42]. We, therefore, analyzed the protein expression levels of common genes related to the aging pathway and found that the deletion of the *Pex3* led to upregulation of the protein expression levels of P19 and P16 and that P16 exerted an inhibitory effect to downregulate the expression level of the downstream protein CDK6 (Fig. 5A/B). In addition, the deletion of PEX3 contributed to the upregulation of the protein level of P53. In addition, P53 inhibited the downstream protein CDK2 to downregulate its expression level. The reductions in the expression levels of CDK2 and CDK6 led to activation (phosphorylation) of the downstream protein Rb [43, 44], causing cell development arrest and apoptosis and ultimately resulting in spermatogenesis disorder. YYH supplementation ameliorated this damage phenotype to a certain extent. Similarly, we found that YYH supplementation attenuated the increase in P16 protein level caused by the deletion of the *Pex3* gene, but it did not significantly affect the protein levels of P19 and P53 (Fig. 5A, B).

### YYH ameliorated the spermatogenesis dysfunction phenotype induced by Pex3-KO through a P16-dependent pathway

**Fig. 6:**
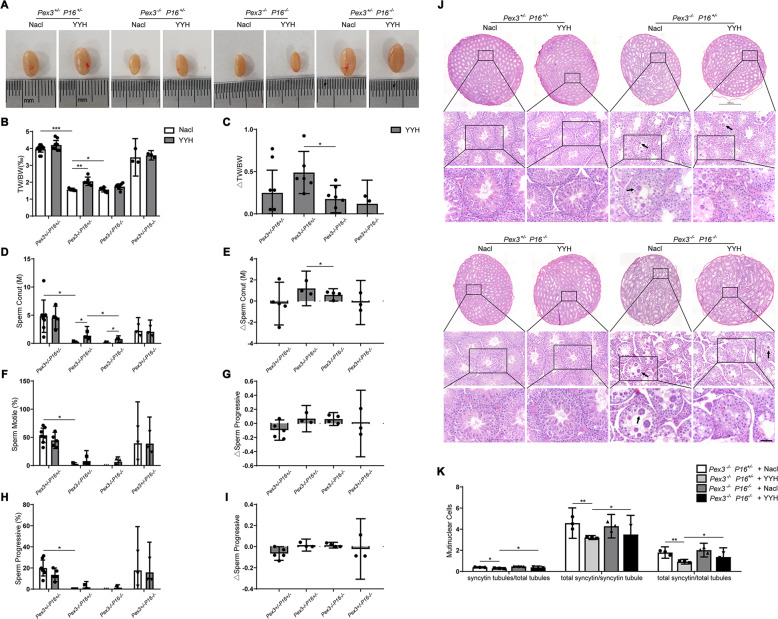
Impacts of YYH treatment on spermatogenesis-related indices in mice with different genotypes (*n* = 4). **A** Representative image of testicular sizes. **B** Testicular weight ratios. **C** Changes in the testicular weight ratio. **D** Sperm counts. **E** Changes in the sperm count. **F** Sperm motility. **G** Changes in sperm motility. **H** Sperm forward motility. **I** Changes in sperm forward motility. **J** Images of HE staining in testes. Scale bars: 50 μm. **K** Statistics regarding syncytial multinucleated cells in the testicular lumen.

As previously mentioned, YYH had a beneficial effect on spermatogenesis in *Pex3*-KO mice. In addition, it significantly inhibited the abnormal increase in the expression of P16 in the absence of PEX3. We consequently hypothesized that the P16-CDK6-Rb signaling pathway mediated the effect of YYH on spermatogenesis in *Pex3*-KO mice. To test this hypothesis, we introduced multiple combinations of gene deletions (Fig. S1). YYH administration groups and normal saline administration groups were included in the experiments (Table 1). By analyzing testicular morphology, testicular weight, sperm counts/motility/progressive motility, and testicular microstructure, we found that YYH administration attenuated the testicular abnormalities caused by *Pex3*-KO (*Pex3*−/−*P16*+/−) and increased the testicular organ ratio. The effect was weakened when *P16* was knocked out (*Pex3*−/−*P16*−/−) (Fig. 6A, B, C). YYH administration also significantly increased the sperm counts of *Pex3*−/−*P16*+/− and *Pex3*−/−*P16*−/− mice (Fig. 6D, E), and the effect was better in the *Pex3*−/−*P16*+/− group. Additionally, YYH administration tended to improve the sperm quality of *Pex3*−/−*P16*+/− and *Pex3*−/−*P16*−/− mice, but the results were not statistically significant (Fig. 6F–I). Furthermore, according to the testicular microstructure analysis, YYH administration markedly reduced the syncytial multinucleated cell abundance of *Pex3*-KO mice but had no significant effect on that of *Pex3*−/−*P16*−/− mice (Fig. 6J, K). Similarly, single deletion of the *P16* gene did not significantly affect spermatogenesis. In conclusion, the ability of YYH to ameliorate spermatogenesis dysfunction phenotypes of *Pex3*-KO mice was reduced when *P16* was knocked out.

**Table. 1:** Design of the experiment.

Group	Genotype	Treat	Dose
I	*Pex3*+/−	Nacl	300 μL
II	*Pex3−/−*	Nacl	300 μL
III	*Pex3*+/−	YYH	325 mg/Kg/day
IV	*Pex3−/−*	YYH	325 mg/Kg/day
V	*Pex3*+/−	TSZ	325 mg/Kg/day
VI	*Pex3−/−*	TSZ	325 mg/Kg/day
VII	*Pex3*+/−	HJT	487 mg/Kg/day
VIII	*Pex3−/−*	HJT	487 mg/Kg/day
IX	*Pex3*+/− *P16*+/−	Nacl	300 μL
X	*Pex3*+/− *P16*+/−	YYH	325 mg/Kg/day
XI	*Pex3−/− P16*+/−	Nacl	300 μL
XII	*Pex3−/− P16*+/−	YYH	325 mg/Kg/day
XIII	*Pex3−/− P16−/−*	Nacl	300 μL
XIV	*Pex3−/− P16−/−*	YYH	325 mg/Kg/day
XV	*Pex3*+/− *P16−/−*	Nacl	300 μL
XVI	*Pex3*+/− *P16−/−*	YYH	325 mg/Kg/day

### The beneficial effect of YYH on IVF outcomes in *Pex3*-KO mice vanished in the absence of *P16*

**Fig. 7:**
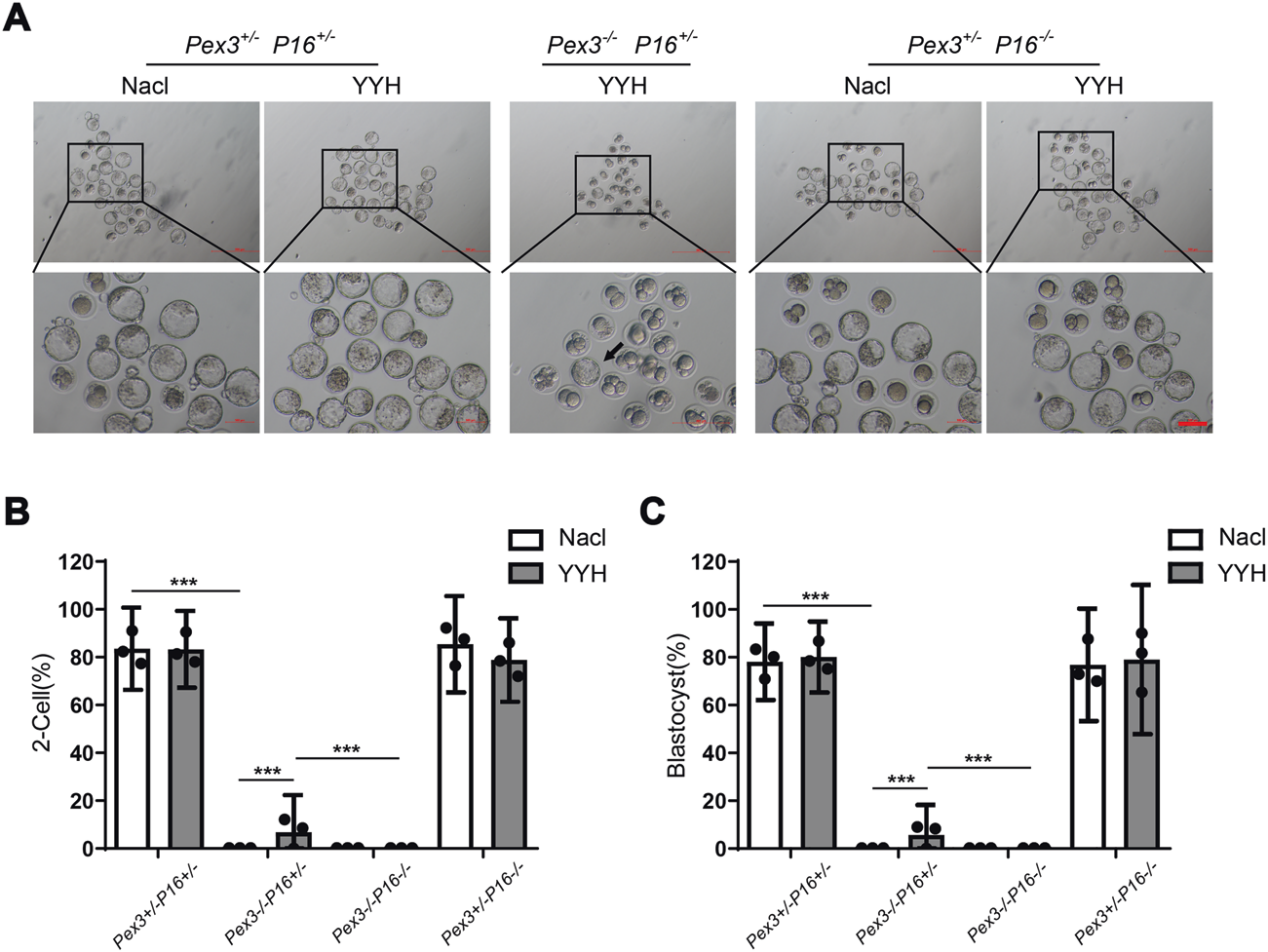
Effects of YYH treatment on early embryonic development in mice with different genotypes. (*n* = 3). **A** Contrast images of blastocyst-stage embryos from each group. Scale bars: 100 μm. **B** Two-cell embryo rate. **C** Blastocyst rate.

We further investigated more comprehensively whether *P16*-KO affected the reproductive outcomes of YYH-treated *Pex3*-KO mice. The results revealed that YYH-*Pex3*−/−*P16*-/− mice had no sperm production and could not undergo IVF. However, early embryos were again able to be successfully obtained from YYH-*Pex3*−/−*P16*+/− mice by IVF (Fig. 7A), and the proportions of embryos that reached the two-cell and blastocyst stages were also remarkably elevated (Fig. 7B, C). These results suggested that YYH improved the reproductive outcomes of *Pex3*-KO mice, thus enabling transplantable embryos to be obtained. However, the effect of YYH on reproductive outcomes disappeared when the *P16* gene was knocked out.

### The beneficial effect of YYH on OS in *Pex3*-KO mice was attenuated in the absence of *P16*

**Fig. 8:**
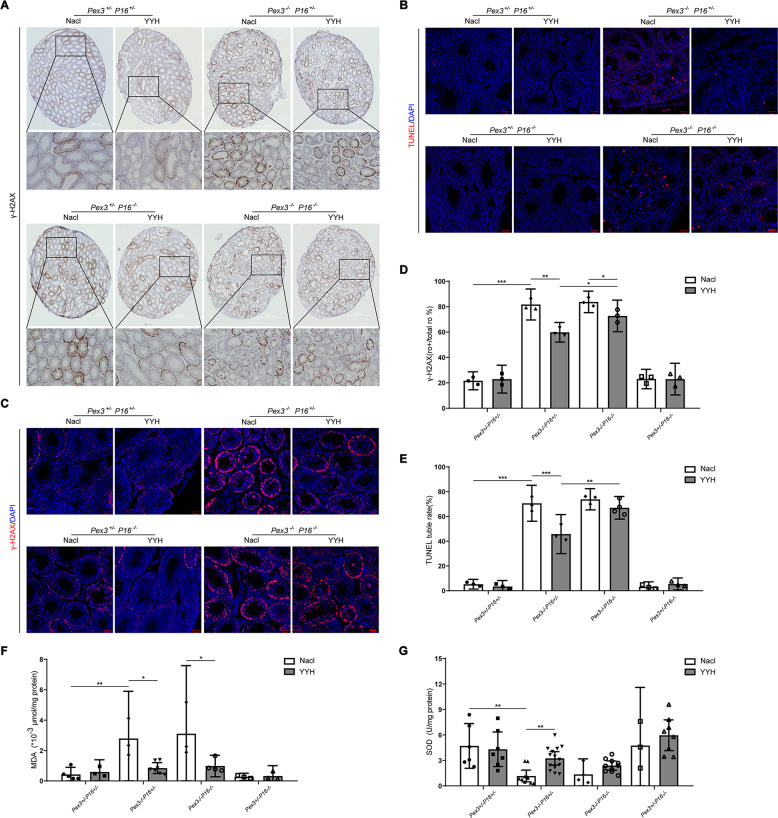
Effects of YYH supplementation on OS levels in testicular tissues of mice with different genotypes. (*n* = 3–6). **A** γ-H2AX immunohistochemistry in testicular sections. Scale bars: 100 μm. **B** TUNEL staining of testicular sections. Scale bars: 50 μm. **C** γ-H2AX immunofluorescence in testicular sections. Scale bars: 50 μm. **D** Rate of γ-H2AX-positive luminal cells. **E** Rate of TUNEL-positive luminal cells. **F** MDA level. **G** SOD level.

We also hypothesized that *P16*-KO affected the antioxidant activity of YYH in *Pex3*-KO mice. We compared the levels of OS-induced DNA damage and apoptosis in testicular tissues of the mice in each group and found that YYH administration ameliorated the peroxidation damage caused by *Pex3* single-gene KO, reduced the numbers of γH2Ax-positive luminal cells (Fig. 8A, C, D) and apoptotic luminal cells (Fig. 8B, E), and decreased the levels of MDA (Fig. 8F) and SOD (Fig. 8G). YYH administration also improved these indices in *Pex3*−/−*P16*−/− mice, but the rescue effects on DNA damage and apoptosis were markedly weaker than those in *Pex3*-KO mice. The above results indicated that when *P16* was knocked out, the ameliorative effect of YYH on OS in *Pex3*-KO mice was observably weakened. Hence, we conclude that YYH is able to exert antioxidant functions through a P16-dependent pathway to improve male infertility caused by *Pex3*-KO.

## Discussion

It is well known that OS is a prominent factor in male infertility or subfertility. Presently, antioxidant supplementation is one of the most common methods of male infertility treatment. TCMs with antioxidant functions, such as Epimedium, is increasingly being used to ameliorate male infertility caused by high levels of OS [45–50]. Nevertheless, due to the complexity of TCM components, most traditional pharmacological studies have focused mainly on the beneficial effect of a single component, such as flavonoids and icariin in YYH. While in practical clinical applications, multicomponent TCMs are mostly used, the precise application of which needs to be further improved. The previous findings inspired us to investigate the efficacy and mechanism of clinical TCMs in order to provide more support for precision medicine.

To achieve this goal, we established mice with *Pex3*-KO. Preliminary studies revealed that *Pex3*-KO led to dyszoospermia and male infertility in male mice, which suggested that the *Pex3*-KO mouse model was a good model with which to study the reproductive effects and mechanisms of antioxidants (Fig. 1). We combined this model with clinical practice to develop a novel approach. Instead of studying a single component, we directly studied the rescue effects of three novel Chinese herbal formula granules with antioxidant functions. The results revealed that YYH supplementation resulted in a small number of spermatids (Fig. 2), and the following IVF study showed that a few transplantable embryos could be obtained from *Pex3*-KO mice (Fig. 3). This result suggests that YYH supplementation may help improve the fertilization potential and the outcome of assisted reproductive technology (ART). More importantly, during in vitro ART treatment, we found that poorer embryo quality was associated with a greater negative paternal contribution. Normally, the relative influence of sperm on the ART outcome is 10–15%. However, an abnormal paternal component strongly affects fertility and the outcome of ART. We, therefore, hypothesize that YYH can be used as the preferred TCM for male infertility with elevated OS to increase the potential of success for male ART patients with severe spermatogenesis disorders [51–53].

In addition, we found increased ROS levels, decreased antioxidant capacity, and elevated DNA damage levels in *Pex3*-KO mice (Fig. 4). Subsequently, a series of age-related phenotypes emerged [54, 55]. We also confirmed that the expression level of P16 and its downstream CDK6 protein had a decreasing tendency (Fig. 5). All of the above results inspired us to study the aging pathway as the breakthrough point [56–58]. YYH supplementation remarkably promoted the phenomenon mentioned above, implying that YYH can indeed improve reproductive function through the ROS-DNA damage-P16-CDK6 signaling pathway. Subsequently, we found that supplementation with YYH did not exert a significant positive effect on young *Pex3*−/−*P16*−/− mice, which indicated that inhibition of the senescence pathway mediated mainly by P16 affected YYH supplementation to some extent even under OS.

Although it would have been common and convenient to further explore YYH’s rescue effect on spermatogenetic blockade related to P16 and the cell senescence pathway using cell lines as models, such a design would not have reflected the real in vivo situation and would have had limited reference value for clinical precision medicine. Hence, we established *Pex3* and *P16* multigene-KO mice (Fig. S1). We found that KO of *P16* weakened the rescue effect of YYH on testicular dysplasia and dyszoospermia caused by *Pex3*-KO (Fig. 6). Moreover, there were no significant rescue effects on DNA damage and apoptosis, and antioxidant activity was weakened (Fig. 8). Similarly, in *Pex3*-KO mice with concurrent *P16*-KO, transplantable embryos could not be obtained after YYH supplementation (Fig. 7). In general, when the *P16* was knocked out, the rescue effect of YYH on reproductive dysfunction caused by *Pex3*-KO was weakened. We speculate that the significant *P16-*KO-induced weakening of the rescue effect of YYH occurred because P16 is a key target of YYH. Additionally, we observed that P16 expression is age-dependent [59, 60], which suggests that although YYH is the preferred TCM for male infertility with increased OS, the effect in young people may not be adequate.

In conclusion, our findings suggest that YYH can reduce ROS levels and DNA damage through antioxidant activity. In addition, YYH regulates the level of P16 protein expression, attenuating excessive P16 protein expression and thereby regulating downstream CDK6 levels, promoting cell proliferation, inhibiting apoptosis, and decreasing abnormal spermatogenic cell arrest. The results show that by mediating ROS-DNA damage-P16-CDK6 pathway signaling, YYH exerts antioxidative effects and can ameliorate clinical male infertility caused by OS and enhance the contributions of male factors in ART. In the future, combining antioxidant supplementation with YYH as the core ingredient with modern ART will open up endless possibilities for male patients with severe infertility.

## Supplementary information

Reproducibility checklist

Figure S1

Figure S2

Figure S3

Figure S4

Figure S5

Supplement Legends

## Data Availability

The data that support the findings of this study are available from the corresponding author upon reasonable request.
