# Supplementary figures and images for: Epimedium protects against dyszoospermia in mice with Pex3 knockout by exerting antioxidant effects and regulating the expression level of P16
Source: Cell Death Dis. 2022 Jan 20;13(1):69. doi: 10.1038/s41419-021-04435-8 (PMC8776794; doi:10.1038/s41419-021-04435-8)

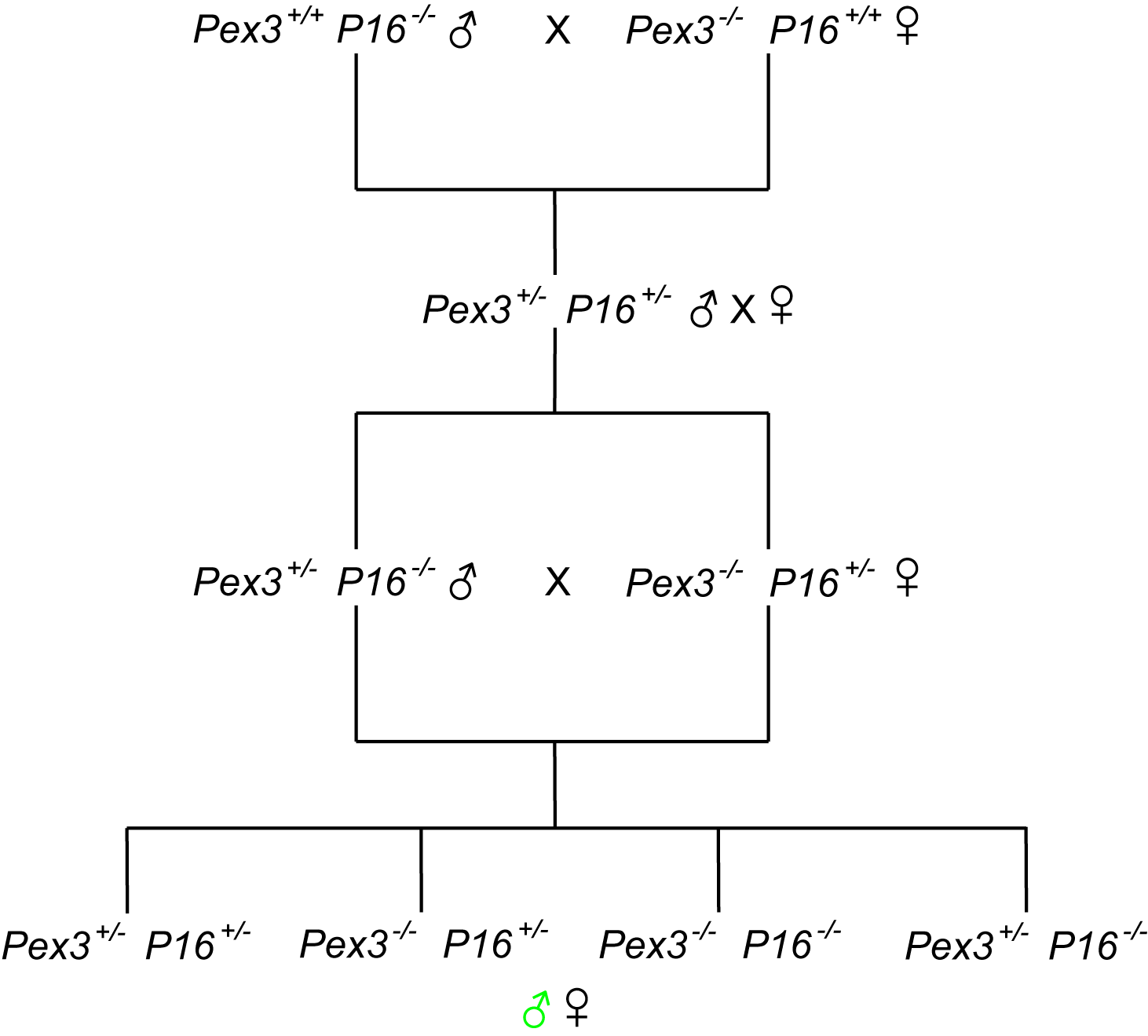

Supplement: Supplementary file 2 — Figure S1 [file 41419_2021_4435_MOESM2_ESM.tif]

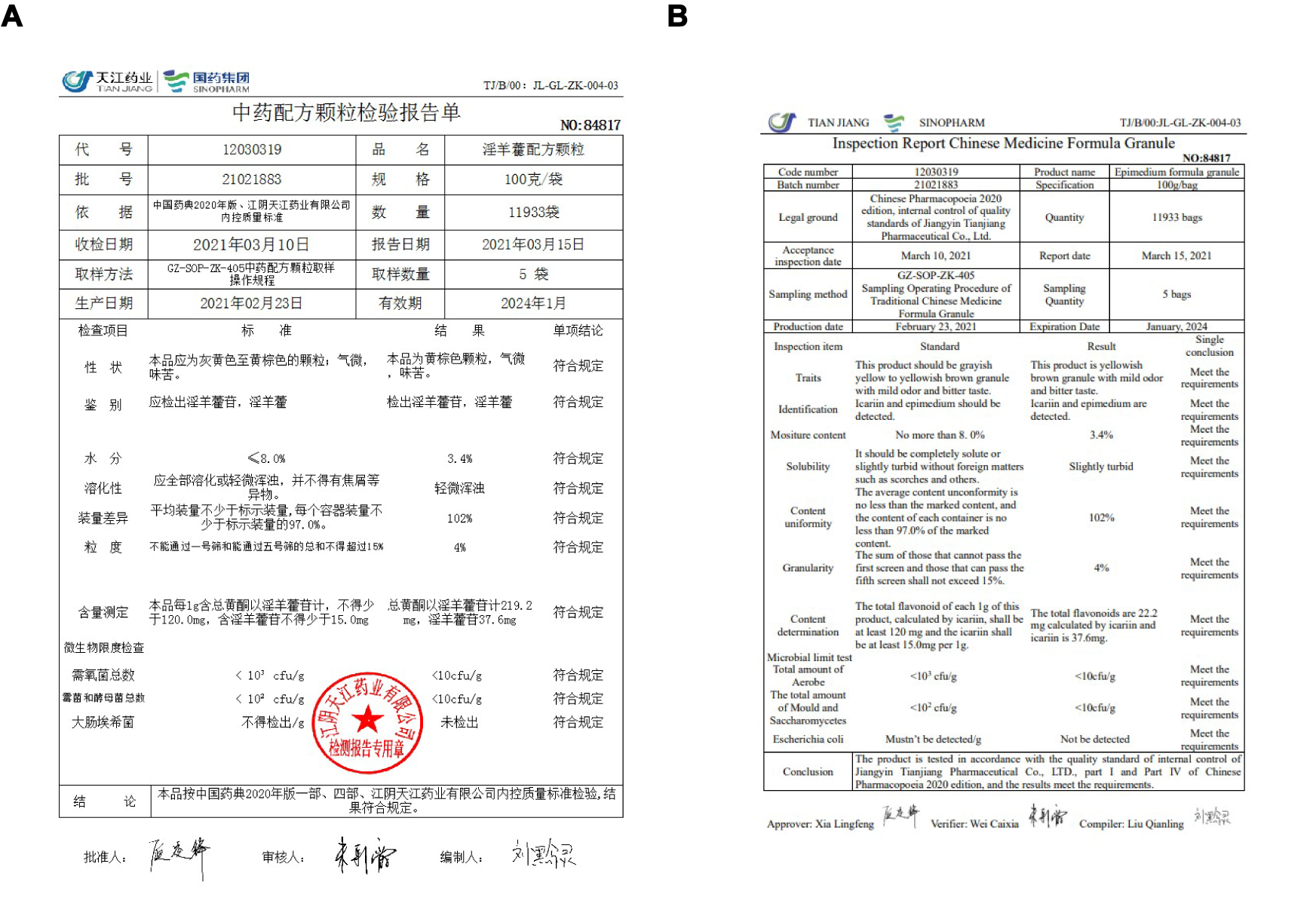

Supplement: Supplementary file 3 — Figure S2 [file 41419_2021_4435_MOESM3_ESM.tif]

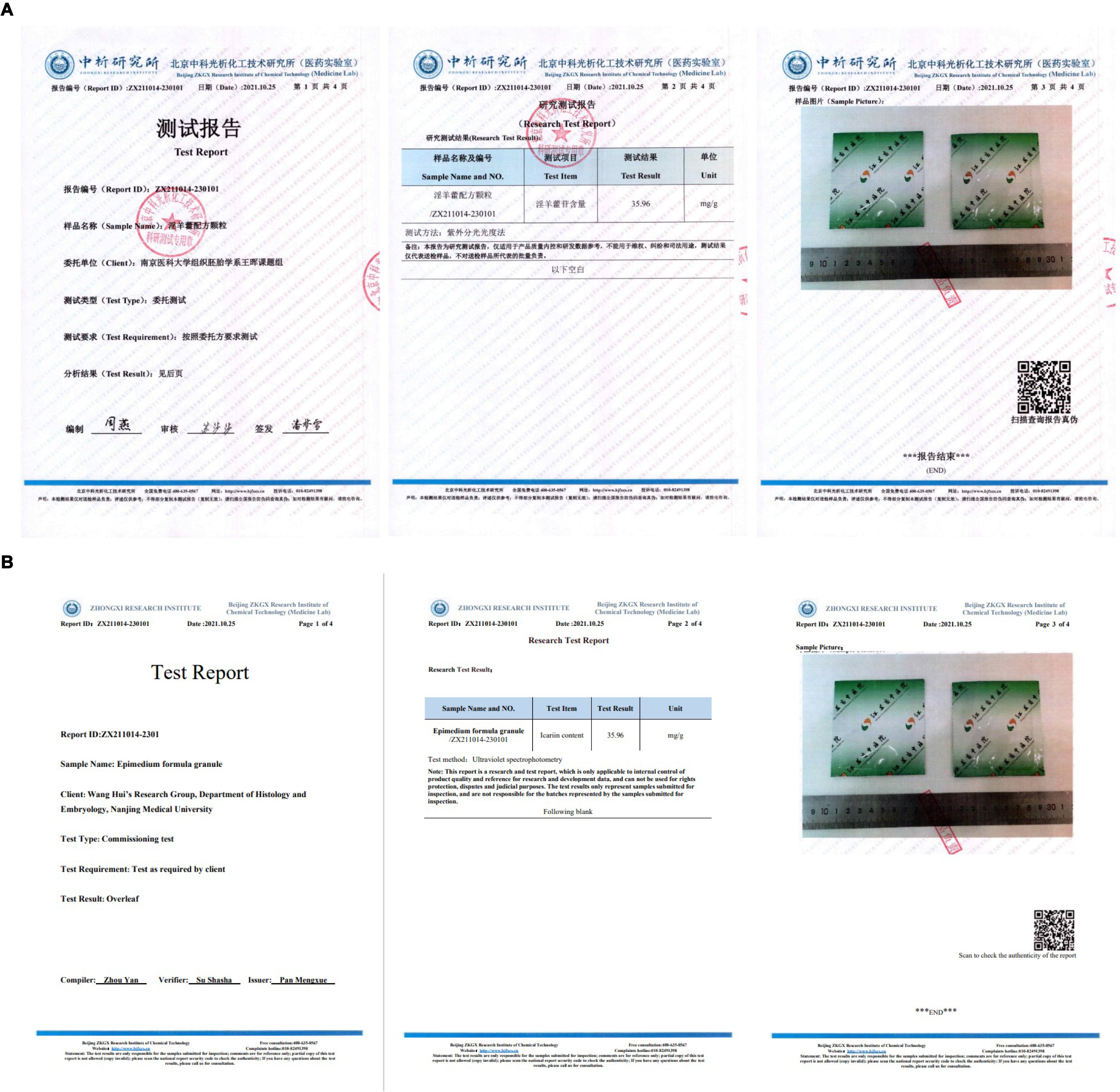

Supplement: Supplementary file 4 — Figure S3 [file 41419_2021_4435_MOESM4_ESM.tif]

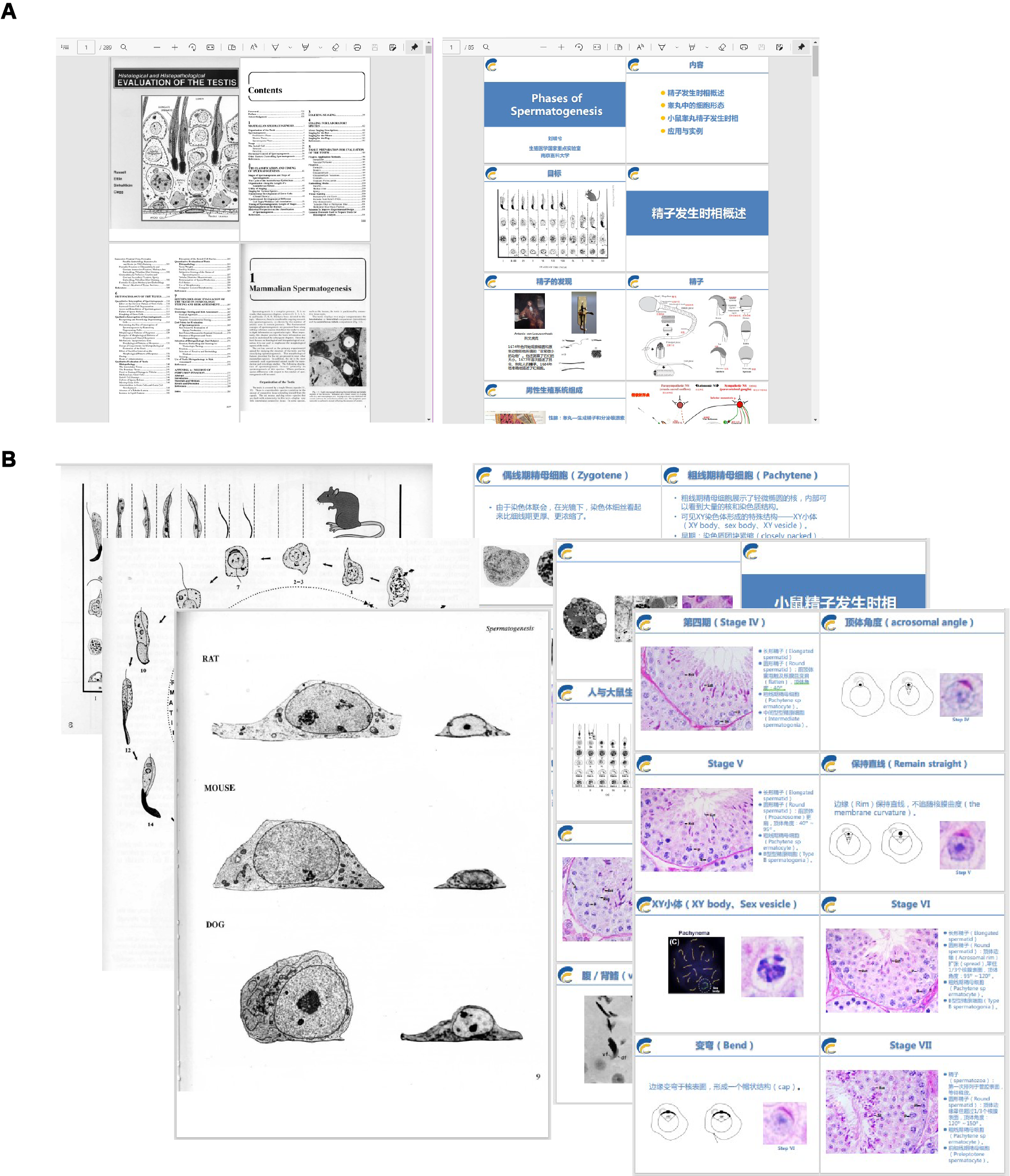

Supplement: Supplementary file 5 — Figure S4 [file 41419_2021_4435_MOESM5_ESM.tif]

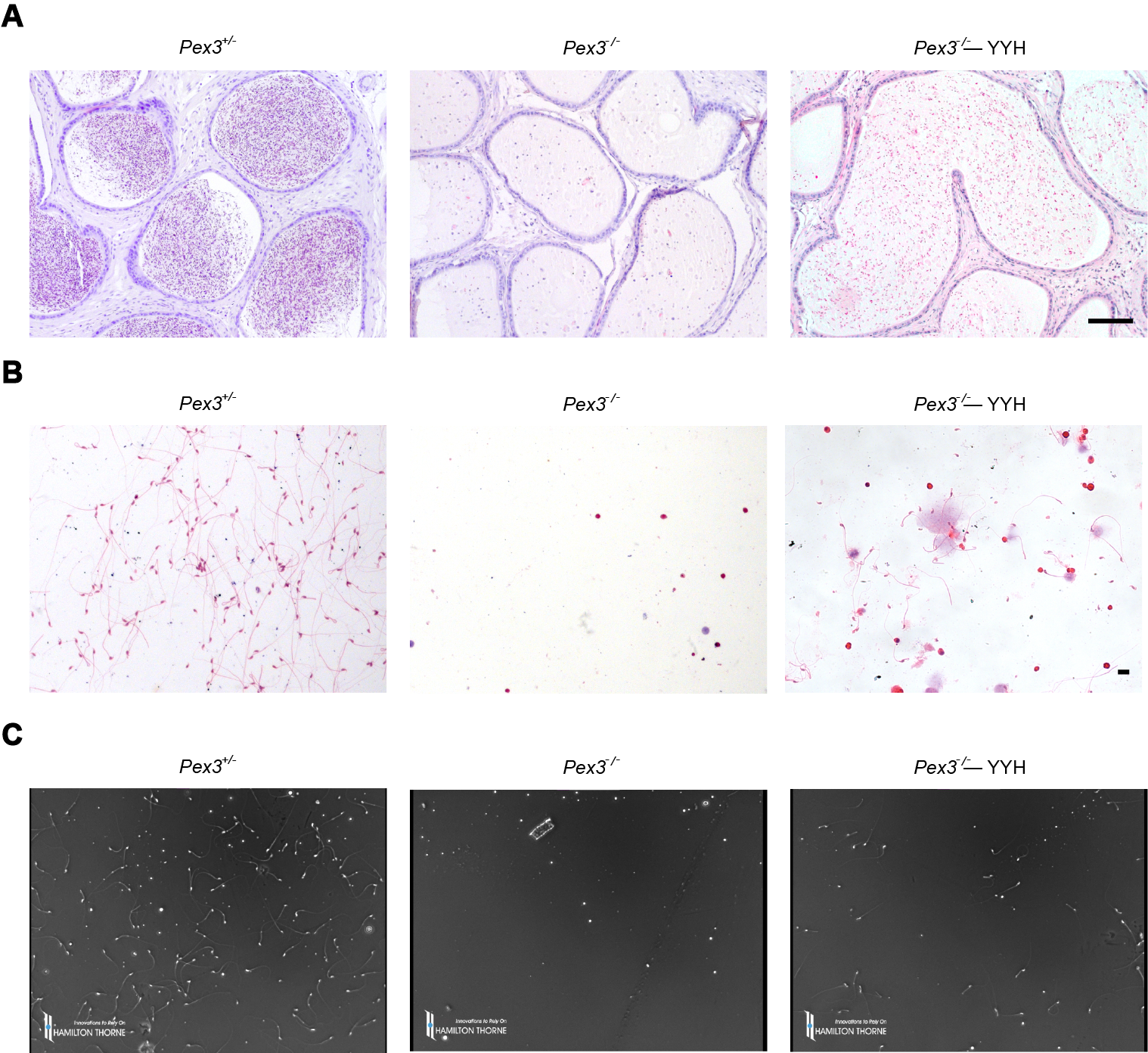

Supplement: Supplementary file 6 — Figure S5 [file 41419_2021_4435_MOESM6_ESM.tif]
