# Supplementary material for: Epimedium protects against dyszoospermia in mice with Pex3 knockout by exerting antioxidant effects and regulating the expression level of P16
Source: Cell Death Dis. 2022 Jan 20;13(1):69. doi: 10.1038/s41419-021-04435-8 (PMC8776794; doi:10.1038/s41419-021-04435-8)
Supplement: Supplementary file 7 — Supplement Legends [file 41419_2021_4435_MOESM7_ESM.docx]

**Supplement Figure Legends**

**Fig. S1** The breeding methods for the mice with complex gene deletion

**Fig. S2** Manufacturer's factory inspection report of YYH

(A) Chinese version.

(B) English version.

**Fig. S3** Third-party detection institution's research test report of YYH

(A) Chinese version.

(B) English version.

**Fig. S4** The training system of PAS experiment

(A) Training syllabus.

(B) The details of the training system.

**Fig. S5** Effects of Epimedium granule supplementation on sperm count of Pex3-KO mice.

(A) HE staining of the epididymal tail. Scale bars: 200 μm.

(B) HE staining of the sperm smear. Scale bars: 20 μm.

(C) The image recorded by the CASA instrument.
